# Are Myocardium Deformation Indices Influenced by Cardiac Load, Age or Body Mass Index?

**DOI:** 10.5935/abc.20190211

**Published:** 2019-10

**Authors:** Vera Maria Cury Salemi, Marcelo Dantas Tavares de Melo

**Affiliations:** 1 Instituto do Coração (InCor) do Hospital das Clinicas da Faculdade de Medicina da Universidade de São Paulo, São Paulo, SP - Brazil; 2 Universidade Federal da Paraíba, João Pessoa, PB - Brazil

**Keywords:** Echocardiography/methods, Myocardium Deformation, Hypertension, Heart Failure, Strain, Hypertrophic Cardiomyopathy

The typical findings of myocardium deformation indices (MDI) in many cardiac diseases, the low cost of the echocardiographic exam, the large availability, the vast implementation of this tool for clinical practice and the prognostic value has allowed the detection of earlier myocardium dysfunction than the traditional measurement of left ventricular (LV) ejection fraction.^1^ Moreover, there are typical MDI patterns in different forms of hypertrophy: decreased values in septum MDI in hypertrophic cardiomyopathy (HCM) or where hypertrophy is more accentuated, or a segmental decrease in mutation carriers in a pre-clinical phase of disease, before development of hypertrophy;^2^ apical sparing in amyloidosis;^3^ striped pattern of myocardium deformation in glycogen storage cardiomyopathy (PRKAG2);^4^ and decrease in subepicardial longitudinal strain in Anderson-Fabry disease.^5^ In hypertension with concentric and eccentric hypertrophy, the MDI patterns are related to different geometric patterns,^6^ but they are frequently preserved in athletes.^4^

The question whether MDI would be reliable in different machines and vendors was demonstrated that the accuracy of these indices were better than conventional echocardiography measurements and they are reliable for daily echocardiographic practice.^7^

The paper published in the same issue of this journal showed that the authors evaluated the LV global longitudinal strain in 45 patients divided into 2 groups: with HCM and the association with hypertension with HCM, and showed that strain was decreased in the latter group compared to the first one.^8^ It is noticeable the difference between both groups about their age, body mass index (BMI) and blood pressure. Besides, many studies have demonstrated that global longitudinal strain could be affected by those variables mentioned, as demonstrated in a paper of 266 healthy subjects, 39.2 ± 17.5 years, 137 women, submitted to transthoracic echocardiography evaluation and showed that global longitudinal strain was progressively reduced with increasing age decades.^9^ Metabolic syndrome may also play a role in myocardial deformation showed in a study of 384 patients grouped according to BMI (normal weight < 25 kg/m^2^, overweight 25-29 kg/m^2^, and obesity ≥ 30 kg/m^2^), compared to healthy control group. Regardless of the presence or not of diabetes, overweight and obesity impair LV ejection fraction and global longitudinal strain.^10^ Interestingly, Russell et al.^11^ were the first to describe a new method that evaluated myocardial work using a non-invasive pressure-strain loop by echocardiogram.^11^ In a canine model, it was demonstrated a significant reduction in LV strain after aortic construction. On the other hand, LV pressure-strain loop area did not change, which means that myocardial work seems to be not affected by increasing the afterload, but global longitudinal strain could transient change by hemodynamics status.^12^ Therefore, we could be more precautious when we stratify the same cardiomyopathy simply by using global longitudinal strain, not considering afterload importance. Furthermore, the previous study compared 80 hypertensive patients, 80 HCM patients and 80 controls showed that longitudinal strain was lower in HCM patients, and also, the best parameter to differentiate both diseases was the MDI ratio of endocardium and epicardium layers. However, this parameter was not evaluated in the present study.^13^

The LV outflow tract obstruction is defined as peak LV gradient greater than or equal to 30 mmHg at rest or with provocation, is present in approximately two-thirds of patients with hypertrophic cardiomyopathy.^14^ This dynamic obstruction leads to an increase in left ventricle afterload which could impair the global longitudinal strain itself, as previous mentioned. However, the authors of this present study did not mention this feature which certainly affects the myocardial deformation. The myocardial thickness and mainly, the presence of fibrosis affect negatively the patients’ prognosis. Both parameters were not described in this present study and it is well known that they are related to a LV global longitudinal strain reduction.^15^ When myocardial work was analyzed in hypertrophic cardiomyopathy subjects, one variable called global constructive work was the only predictor of LV fibrosis at multivariable analysis (OR 1.01, 95% CI: 0.99 - 1.08, p = 0.04).^16^ A cutoff value of 1623 mmHg% (AUC 0.80, 95% CI: 0.66-0.93, p < 0.0001) was able to predict myocardial fibrosis with good sensitivity and fair specificity (82% and 67%, respectively).

In conclusion, MDI are an important tool helping to distinguish HCM from other cardiomyopathies, as well as, they present value for risk stratification impact. However, it is extremely recommended to take into account the hemodynamic status whenever analyze MDI data. Moreover, it is a diagnostic challenge the presence of overlapping of hypertrophic cardiomyopathy and hypertension. Myocardial work may play a role to solve this.
